# Strain specific differences in vitamin D3 response: impact on gut homeostasis

**DOI:** 10.3389/fimmu.2024.1347835

**Published:** 2024-03-01

**Authors:** Laura Schreiber, Sakhila Ghimire, Andreas Hiergeist, Kathrin Renner, Michael Althammer, Nathalie Babl, Alice Peuker, Gabriele Schoenhammer, Katrin Hippe, Andre Gessner, Christin Albrecht, Fransziska Pielmeier, Maike Büttner-Herold, Heiko Bruns, Petra Hoffmann, Wolfgang Herr, Ernst Holler, Katrin Peter, Marina Kreutz, Carina Matos

**Affiliations:** ^1^ Department of Internal Medicine III, Hematology and Medical Oncology, University Hospital Regensburg, Regensburg, Germany; ^2^ Institute of Clinical Microbiology and Hygiene, University Hospital Regensburg, Regensburg, Germany; ^3^ Department of Otorhinolaryngology, University Hospital Regensburg, Regensburg, Germany; ^4^ Department of Pathology, University of Regensburg, Regensburg, Germany; ^5^ Leibniz Institute for Immunotherapy (LIT), Regensburg, Germany; ^6^ Department of Nephropathology, Institute of Pathology, Friedrich-Alexander-University Erlangen-Nürnberg (FAU), Erlangen, Germany; ^7^ Department of Internal Medicine 5, Hematology and Oncology, Friedrich-Alexander-University Erlangen-Nürnberg (FAU), Erlangen, Germany

**Keywords:** vitamin D3, barrier function, intestinal, C57BL/6, BALB/c

## Abstract

Vitamin D3 regulates a variety of biological processes irrespective of its well-known importance for calcium metabolism. Epidemiological and animal studies indicate a role in immune regulation, intestinal barrier function and microbiome diversity. Here, we analyzed the impact of different vitamin D3- containing diets on C57BL/6 and BALB/c mice, with a particular focus on gut homeostasis and also investigated effects on immune cells *in vitro*. Weak regulatory effects were detected on murine T cells. By trend, the active vitamin D3 metabolite 1,25-dihydroxyvitamin D3 suppressed IFN, GM-CSF and IL-10 cytokine secretion in T cells of C57BL/6 but not BALB/c mice, respectively. Using different vitamin D3-fortified diets, we found a tissue–specific enrichment of mainly CD11b+ myeloid cells but not T cells in both mouse strains e.g. in spleen and Peyer’s Patches. Mucin Reg3γ and Batf expression, as well as important proteins for gut homeostasis, were significantly suppressed in the small intestine of C57BL76 but not BALB/c mice fed with a high-vitamin D3 containing diet. Differences between both mouse stains were not completely explained by differences in vitamin D3 receptor expression which was strongly expressed in epithelial cells of both strains. Finally, we analyzed gut microbiome and again an impact of vitamin D3 was detected in C57BL76 but not BALB/c. Our data suggest strain-specific differences in vitamin D3 responsiveness under steady state conditions which may have important implications when choosing a murine disease model to study vitamin D3 effects.

## 1 Introduction

Besides its classical role in maintaining calcium and bone metabolism vitamin D3 has been associated with the regulation of immune responses and the maintenance of intestinal homeostasis (1). To become active, vitamin D3 needs to go through two consecutive hydroxylation steps. In the first step, calcidiol or 25(OH)D3 is produced in the liver, while the active metabolite, calcitriol or 1,25(OH)2D3 is mainly produced in the kidney. The biological activity of 1,25(OH)2D3 is mediated by the vitamin D receptor (VDR) (1).

The role of vitamin D3 metabolites in modulating immune responses is well acknowledged. Vitamin D3 metabolites promote a tolerogenic phenotype in dendritic cells (2–4), which in turn can modulate T cell responses. In addition, T cells are direct targets of vitamin D3 metabolites and it has been shown that especially 1,25(OH)2D3 can suppress Th1 and Th17 responses, while enhancing Th2 and regulatory T cells (Tregs) (5–8).

The impact of vitamin D on intestinal homeostasis is highlighted in epidemiological studies that document an association between calcidiol/25(OH)D3 deficiency and increased risk of inflammatory bowel disease (IBD), such as Colitis and Crohn’s Disease (9, 10). Since the VDR is highly expressed in gut epithelial cells (11) it makes it an attractive therapeutic target in controlling inflammatory bowel conditions. Furthermore, several studies demonstrated that vitamin D3 and the VDR influence microbiome composition both in humans and in mice (12–15). In a genome-wide association study, Wand and colleagues found that human VDR gene variations correlate with changes in the intestinal microbiome (13). In mice, the lack of VDR and its relation to the microbiome was demonstrated by several groups (14–16), with VDR KO mice displaying dysbiosis (14). In addition, VDR KO mice exhibit altered Paneth cell function resulting in defective antimicrobial peptide secretion and autophagy (17, 18).

Transplantation and tumor immunology research is often conducted in mouse models, with C57BL/6 and BALB/c being the most frequently used mouse strains. It is known that these two mice strains differ in their immunological responses. In a study by Scott and Farrell (19), the authors observed that while C57BL/6 mice are resistant to leishmanial major infections, BALB/c mice died after infection with the same pathogen. In a different work, using models for Mycobacterium tuberculosis-infection, Bertolini et al. (20) demonstrated that infected C57BL/6 mice generate a stronger cellular immune response compared with BALB/c mice. Furthermore, it is known that C57BL/6 mice are prone to generate a Th1- and M1-dominant immune response, while BALB/c mice show a more Th2- and M2-dominant immune response (21). Based on the observed immunological differences between the two mouse strains, we sought to investigate the impact of different vitamin D3- containing diets in both C57BL/6 and BALB/c, with particular focus on gut homeostasis and the microbiome.

## 2 Materials and methods

### 2.1 Animals

All animal experiments were performed according to the regulations of the local government located in Würzburg, Germany. Both C57BL/6N and BALB/c female mice were purchased from Charles River. The mice were randomly allocated to the different diets and cages. Each diet consisted of 2 cages of mice, 5 mice/cage. In total, 10 mice per group were used. The mice were fed with different vitamin D3- containing diets (SSNIFF Spezialdiäten GmbH) directly from weaning until the age of 12 to 15 weeks.

### 2.2 Diet composition

All purified diets were purchased from SSNIFF Spezialdiäten GmbH. They were based on the vitamin D deficient diet and vitamin D was supplemented to reach a “low/marginal” level (400 IU/kg), “normal” level (1500 IU/kg) and “high” level (5000 IU/kg). The Ca and P content was kept on a normal level for all 3 diets. A more detailed composition can be found in **Table 1**.

**Table 1:** Composition of the diets given to the animals in the present study.

Dietary composition	Low Vitamin D3	Normal Vitamin D3	High Vitamin D3
Vitamin D3*	**400 IU**	**1500 IU**	**5000 IU**
Casein	240	240	240
Corn starch	300	300	300
Maltodextrin	195	195	195
Dextrose	100	100	100
Cellulose powder	50	50	50
L-Cystine	2	2	2
Vitamin mix – Cholecalciferol free	10	10	10
Mineral & trace element mix	60	60	60
Choline chloride	2	2	2
Soybean oil	40	40	40
Dye	0.40	0.40	0.40

*Vitamin D3 is given in IU/kg. The remaining components are presented in g/kg.

### 2.3 25(OH)D3 determination in serum

Blood was collected from the tail vein and allowed to set for 20 minutes at room temperature. Afterwards, the samples were centrifuged at 10000 g for 15 minutes. Serum was carefully collected, transferred to a new Eppendorf-tube and stored at -20°C until analysis. 25(OH)D3 levels were analysed by means of liquid chromatography high-resolution tandem mass spectrometry as described elsewhere (22).

### 2.4 Tissue preparation for flow cytometry analysis

#### 2.4.1 Murine splenocytes

Mice spleens were scratched out to gain splenocytes in a single-cell suspension and erythrocytes were removed with ACK lysis buffer (0.155 M NH4Cl, 0.1 M KHCO3 and 0.1 mM EDTA, diluted 1:6 with H2O).

#### 2.4.2 Bone marrow

Single cell suspensions were obtained from both femur and tibia of mice by flushing out the bone marrow using a 27-gauge syringe filled with ice-cold PBS. After smashing the bone marrow through a cell strainer, cells were centrifuged and erythrocytes removed with ACK lysis buffer.

#### 2.4.3 Intestinal leukocytes

Small and large intestines were excised, Peyer’s patches removed, cut into 1–3 cm pieces, incubated twice for 15 min at 37 °C in HBSS/5mM EDTA/1mM DTT (Sigma-Aldrich, Munich, Germany) then vigorously shaken to isolate intraepithelial leukocytes (IEL). Peyers´s patches were smashed through a cell strainer and washed with ice-cold PBS. Afterwards the cells were centrifuged and the pellet re-suspended in RPMI medium. For lamina propria leukocytes (LPL), the fragments were transferred to a new falcon tube and the Lamina Propria Dissociation Kit from Miltenyi Biotec was used. Briefly, the pieces were digested using warm digestion solution, incubated at 37 °C for 30 min and further dissociated using GentleMACS® (Miltenyi Biotec). Afterwards, the cells were strained and washed before proceeding to flow cytometry staining.

### 2.5 Flow cytometry

Single cell suspensions were generated from murine tissues of C57/BL6 and BALB/c mice. Afterwards, cells were treated with FcR-blocking reagent (Miltenyi Biotec) for 10 min at 4°C after washing with flow cytometry buffer. To exclude dead cells, samples were washed twice with PBS and incubated with BD Horizon™ Fixable Viability Stain 510 viability dye (for 10 min at RT in the dark). Subsequently surface staining to discriminate immune cell populations with PerCP anti-CD45 (30-F11, BD Biosciences), APC-Cy 7 anti-TCRβ (H57-597, BioLegend), Pe-Cy7 anti-CD11b (M1/70, BD Biosciences), and anti-CD19 (6D5, BioLegend), was performed. The data was acquired using a BD FACS LSR II instrument (BD Biosciences) and analyzed with FlowJo software (Tree Star Inc, version 9.9.6 or 10.4.1).

### 2.6 *In-vitro* analysis of CD4^+^ T cells

CD4^+^ T cells were isolated from spleens by magnetic bead separation (Miltenyi) and cultured in RPMI 1640 Medium containing Pen/Strep, 10%FCS, 2 mM Glutamine and 100 IU/ml IL-2. T cells were stimulated with plate-bound anti-CD3 and soluble anti-CD28 in the absence or presence of 5 nM 1,25(OH)2D3 (Cayman Chemical) for 3 days.

### 2.7 Determination of cytokines in the culture supernatants

Determination of cytokines in culture supernatants was performed using ELISA kits from R&D Systems (Minneapolis, MN, USA).

### 2.8 Tissue preparation for RNA and Immunohistochemistry

Colon and small intestine were retrieved from mice and immediately washed with cold PBS. Any attached adipose tissue was removed and a cotton swab was used to empty the colon from remaining stool. Afterwards, both tissues were cut longitudinally and half was placed in a tissue-processing/-embedding cassette. Subsequently, the cassettes were placed in 10% formalin overnight at room temperature. The remaining half of the tissue was cut in small pieces, placed in RNAlater Stabilization Solution (ThermoFisher) and stored at -20°C until analysis.

### 2.9 Preparation of RNA, reverse transcription and quantitative realtime PCR

Total cellular RNA was extracted using the RNeasy Mini Kit (Qiagen, Hilden, Germany). RNA concentration was measured using a NanoDrop Spectrophotometer (Thermo Fisher Scientific, Schwerte, Germany). Reverse transcription was performed with 500 ng RNA in a total volume of 20 μl using an M-MLV Reverse Transcriptase from Promega (Mannheim, Germany). For reverse transcription quantitative real-time PCR, 1 μl cDNA, 0.5 μl of primers (10 μM) and 5 μl QuantiFast SYBR Green PCR Kit(Qiagen) in a total of 10 μl were applied. Primer sequences (all from Eurofins MWG Operon, Ebersberg, Germany) were as follows (−5′-3′); (F- Forward; R- Reverse): *Vdr*_F: AGATCTGTGAGTCTTCCCAGGAGAG; *Vdr*_R: GAGGCACATTCCGGTCAAAGTCAC, *Cyp27b1*_F: CGTGCTTGCGGATTGCTAACGG; *Cyp27b1*_R: TAGTCGTCGCACAAGGTCACGG; *Cyp2r1*_F: GGGTGAACTCATCATTGCTGGAACTG; *Cyp2r1*_R: TGTATTCCCAAGAAGGTCTCCTGTTGTG; *Muc2*_F: CGTGAGGATGATGCCCAT TGAG; *Muc2*_R: GACAGAAGCAGCCTTCCACC; *Reg3γ*_F: ATGGCTCCTATTGCTATGCC; *Reg3γ*_R: GATGTCCTGAGGGCCTCTT; *Batf_*F: GGAAGATTAGAACCATGCCTC; *Batf*_R: CCAGGTGAAGGGTGTCGG; *18S_rRNA_F:* ACCGATTGGATGGTTTAGTGAG; *18S_rRNA_R: CCTACGGAAACCTTGTTACGAC*.

### 2.10 Immunohistochemistry

Tissue samples were obtained after sacrificing mice. Samples were immediately transferred to formalin and embedded in paraffin. Formalin fixed paraffin-embedded (FFPE) samples were cut 2-3 µm thick and were placed on Superfrost microscopic slides. Slides were dried on a hot plate at 70°C for 30 min, deparaffinised in xylol and rehydrated in descending alcohol line. Antigen retrieval was performed with citrate buffer (pH 7.2) in a microwave at 320 watt for 35 minutes. Endogenous peroxidase was blocked with peroxidase blocking solution (DAKO, S2023) for 5 minutes at room temperature (RT). Slides were incubated with rabbit monoclonal anti-VDR antibody (clone D2K6W, CellSignaling) at the dilution of 1:100 for 1 hour at RT. HRP-conjugated secondary antibody (Histofine, 414334F) was added to the slides for 45 minutes at RT. Slides were incubated with DAB substrate for 10 minutes at RT and were counterstained with haemalaun. Slides were dehydrated in ascending alcohol line followed by Xylol. Tissue sections were covered with cover slip using entellan. The tissue slides were scanned with Pannoramic Scanner 250 (Sysmex) at 20X magnification and were evaluated with CaseViewer (Sysmex). PAS- staining was used for Goblet cell evaluation.

### 2.11 Quantification of VDR immunohistochemistry signal

The staining score was calculated as a product of multiplication between positive cells proportion score (0-4) and the staining intensity score (0-3).

### 2.12 Microbiome analysis

#### 2.12.1 Extraction of microbial DNA and 16S rDNA high-throughput ampicon sequencing

Microbial DNA was extracted from 25 mg of fresh fecal boli, which have been stored at -80°C upon processing. Stool suspensions were pre-treated by repeated bead beating using the Lysing Matrix Y beads (MP Biomedicals, Solon, OH, USA) on a TissueLyzer II (Qiagen, Hilden, Germany) followed by purification of nucleic acids from fecal lysates with the MagNA Pure 96 system (Roche Diagnostics, Rotkreuz, Switzerland). Bacterial 16S rDNA copy numbers in the extracted DNA were determined as per the method described by Staemmler et al. (23). Specifically, total bacterial 16S rRNA gene copy numbers in the isolated DNA were quantified using qPCR on the LightCycler 480 II Instrument (Roche Diagnostics, Rotkreuz, Switzerland). The PCR reactions included universal eubacterial 16S rRNA gene primers 764F and 907R, along with the LightCycler 480 SYBR Green I Master kit (Roche Diagnostics, Rotkreuz, Switzerland). For standard quantification, complex PCR amplicon mixtures of full-length 16S rRNA genes from human fecal DNA have been cloned into pGEM TEasy (Invitrogen, Thermo Fisher Scientific, Waltham, MA, USA). Microbiome sequencing was conducted in a DIN EN ISO 15189 accredited workflow. Specifically, V3-V4 variable regions of the 16S rRNA gene were separately amplified from nucleic acid extracts normalized to 1E7 16S rDNA copies for each sample using universal primer pairs 341F/815R. The barcoded PCR products across all samples were pooled and purified using AmpureXP Beads (Beckman Coulter, Indianapolis, IN, USA). The sequencing library’s quantification was performed using the Ion Library TaqMan™ Quantitation Kit, and the resultant amplicons were sequenced using the Ion GeneStudio S5 Plus system (Thermo Fisher Scientific, Waltham, MA, USA).

#### 2.12.2 Processing of sequencing reads and generation of zOTUs

The raw sequencing data, initially obtained from Torrent Suite 5.18, underwent further processing with cutadapt 4.1 (24) for the removal of adapters and primers, as well as demultiplexing. This was followed by sequence filtering, employing a quality threshold of 15 within a 10-base sliding window using Trimmomatic 0.39. Any DNA sequences shorter than 250 bases were excluded. The quality-filtered sequencing data was then processed through a pipeline based on vsearch 2.26.1. Reads exhibiting more than five expected errors were discarded. zOTUs (zero-radius operational taxonomic units) were constructed from the quality-filtered reads, adhering to an alpha value of 2 and a minimum threshold of 5 reads. The uchime3_denovo algorithm was used to eliminate chimeric sequences. Reads displaying 98 percent pairwise identities were realigned to non-chimeric zOTUs using the usearch_global algorithm. Taxonomic assignment was carried out in R 4.3.2, utilizing the IDTAXA classifier from DECIPHER 2.26.0 (25) and the Genome Taxonomy Database (GTDB, release 214.1) (26). A 98 percent bootstrap cutoff was employed for tree traversal, and taxonomic identification at each level was reported with a confidence threshold of 40.

#### 2.12.3 Statistical analysis

Statistical analyses and graph creations were conducted using R (version 4.3.2) within the RStudio environment. In this study, bacterial alpha diversity was quantified using the Inverse Simpson index through the mia package (version 1.1.7). Relative Abundance plots were generated with microeco 1.4.0 (27). Linear mixed effects models were applied to alpha diversity measures using restricted maximum likelihood (REML) methodology, employing lmerTest::lmer (lmerTest, version 3.1–3), and incorporating random slopes and intercepts according to the model design. Vitamin D3 dosage, study timepoint and mouse strains were used as fixed effect, individual mice were used as a random effect in the formula to account for repeated sampling effects. Statistical inference was achieved through likelihood-ratio tests using lmerTest::anova. Furthermore, *post-hoc* pairwise testing was carried out using emmeans::emmeans (emmeans, version 1.8.4–1). Differential abundances between treatment groups, timepoints and mouse strains were examined using two distinct methods within the full Linear Mixed Effects (LME) framework. For MicrobiomeStat::LinDA (version 1.1), features with a mean abundance below 0.1 percent across all samples and a prevalence below 1 percent were filtered, considering zOTUs below an alpha value of 0.1 as significant.

## 3 Results

### 3.1 1,25(OH)2D3 differentially modulates cytokine secretion in murine T cell cultures, depending on the mouse strain

**Figure 1:**
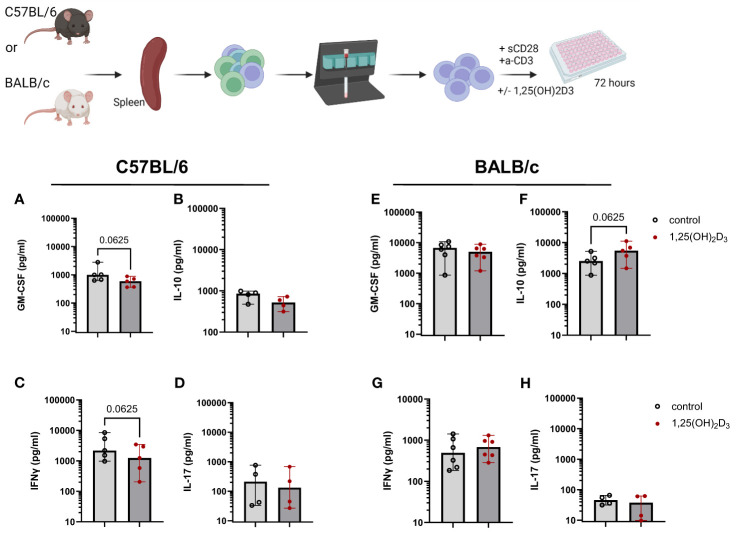
1,25(OH)2D3 differentially modulates cytokine secretion in murine T cell cultures, depending on the mouse strain. CD4+ T cells were isolated either from C57BL/6 or BALB/c splenocytes using MACS technology. Afterwards, the cells were cultured in RPMI medium supplemented with 10% FCS serum and 100 IU/ml IL-2. The cells were activated using plate-bound anti-CD3 and soluble anti-CD28 for 72 hours in the presence or absence of 5 nM 1,25(OH)2D3. The supernatants were then harvested and stored at -20°C until further analysis. The amount of GM-CSF **(A, E)** IL-10 **(B, F)**, IFNγ **(C, G)** and IL-17 **(D, H)** were determined by means of ELISA. Each dot represents one individual mouse. Bars indicate the median. Statistical analysis was performed using Wilcoxon test. Upper panel created with Biorender.com

A large body of research on vitamin D3 has shown the immunomodulatory function of this hormone. We and others (7, 8) found that 1,25(OH)2D3 significantly decreased IFN-γ secretion in human T cell cultures, while increasing IL-10 and sCTLA4. Therefore, in a first step, we investigated the impact of the active metabolite, 1,25(OH)2D3 in murine T cell cultures *in vitro*. For that, CD4^+^ T cells were isolated from the spleen of both C57BL/6 and BALB/c WT mice, both fed with the normal 1500 IU/Kg Vitamin D3 (VD3) diet, and cultured in the presence or absence of 5 nM 1,25(OH)2D3. The two mouse strains reacted differently to the addition of 1,25(OH)2D3 to the cultures. Overall, comparing the two strains, higher levels of GM-CSF and IL-10 were detected in supernatants of BALB/c compared to C57BL/6 T cells cultures. However, IFNγ and IL-17 seem to be secreted in higher amounts in cultures derived from C57BL/6 mice. GM-CSF secretion was decreased upon 1,25(OH)2D3 addition for C57BL/6 mice, although this difference did not reached statistical significance. A similar trend was observed for BALB/c mice (**Figures 1A, E**). Surprisingly, we also observed a decreased secretion of IL-10 in C57BL/6 mice upon 1,25(OH)2D3 treatment (**Figure 1B**), but an opposite effect in T cells from BALB/c mice (**Figure 1F**). IL-17 secretion was rather low and not significantly modulated in both mouse strains (**Figures 1D, H**). In accordance with the literature, IFNγ secretion was decreased upon 1,25(OH)2D3 treatment (**Figures 1C, G**). However, this was only observed in C57BL/6 cultures, while CD4^+^ T cells isolated from BALB/c splenocytes did not react in the same fashion (**Figure 1H**). These data suggests that the two mouse strains react differently to vitamin D3 treatment.

### 3.2 25(OH)D3 serum levels of C57BL/6 and BALB/c mice fed with different vitamin D3- containing diets

**Figure 2:**
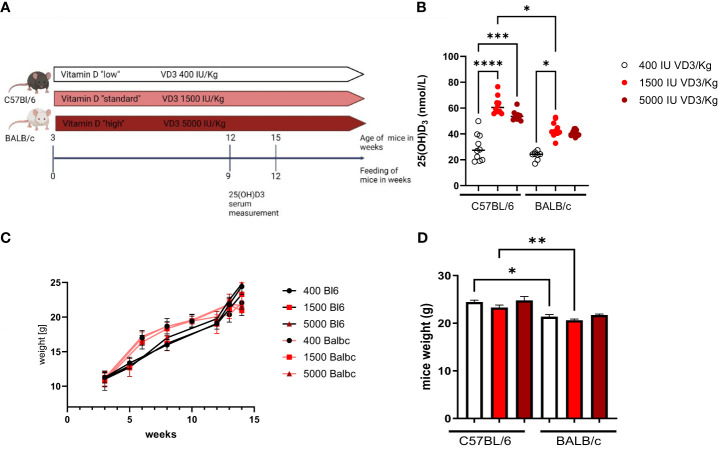
The study design is presented in **(A)**. (Figure created with Biorender.com) In **(B)** 25(OH)D3 serum levels of C57BL/6 and BALB/c mice fed with different vitamin D3- containing diets are shown. Mice were fed directly from weaning until 12 weeks of age, either with Vitamin D low (400 IU/kg) standard (1500 IU/kg) or high (5000 IU/kg) diets. Blood samples were collected from the animal's tail vein. Afterwards, the sample was centrifuged and the serum collected and frozen until analysis. On **(C)** the weight evolution of the mice during the study is presented. The graph bar in **(D)** represents the weight of the mice at the end of the study. Displayed is the median, each symbol represents an individual mouse. Statistical analysis was performed using one-way ANOVA, followed by Kruskal-Wallis test (*p ≤ 0.05, **p ≤ 0.01, ***p ≤ 0.001; **** p<0,0001).

Due to the observation that T cells isolated from C57Bl/6 or BALB/c mice responded differently to 1,25(OH)2D3 treatment *in vitro*, we sought to investigate the impact of dietary vitamin D3 in the two mouse strains. For this purpose, female C57BL/6 and BALB/c mice were fed different diets directly from weaning until the age of 12 to 15 weeks. Since a “normal” diet given to a laboratory mouse contains around 1500 IU/kg VD3, we investigated the impact of a lower VD3 (400 IU/kg) and a higher VD3 (5000 IU/kg) supplementation. At the age of 12 weeks, blood was collected from the tail vein and the serum collected after centrifugation (**Figure 2A**). As observed in **Figure 2B**, a diet containing 1500 IU/kg VD3 led to higher 25(OH)D3 serum levels compared to mice fed a VD3 low diet in both mouse strains. However, C57BL/6 mice reached higher levels compared with BALB/c mice. Although the mice were fed with 5000 IU VD3 as well, this higher VD3 level in food was not reflected in higher 25(OH)D3 serum levels. Regarding the impact of the diets on mouse weight, we observed no difference between the diets. However, for diets with 400 and 1500 IU VD3/kg we observed that C57BL/6 mice were significantly heavier compared with BALB/c (**Figures 2C, D**).

### 3.3 Immune cell composition in blood, spleen and gut of C57BL/6 and BALB/c mice

**Figure 3:**
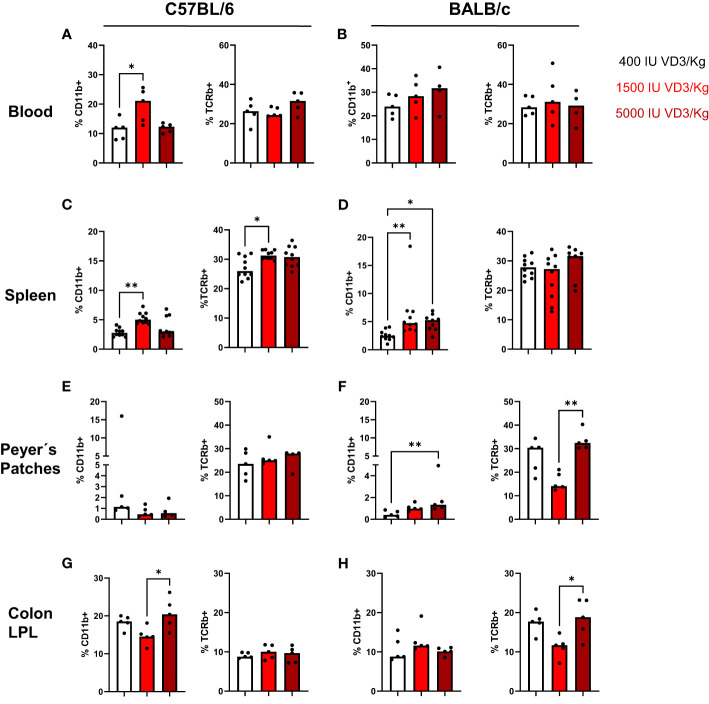
Vitamin D modulates the immune cell composition living in blood, spleen and gut of C57BL/6 and BALB/c mice. Quantification of myeloid cells (left) and T cells (right) in C57BL/6 and BALB/c mice among Iiving CD45+ mononuclear cells in the blood **(A, B)**, spleen **(C, D)**, peyer's patches **(E, F)** and colon lamina propria leucocytes **(G, H)** of animals fed with different vitamin D3 diets. Displayed is the median. Each dot represents one individual mouse Statistical analysis was performed using one-way ANOVA, followed by Kruskal-Walls test (*p ≤ 0.05, **p ≤ 0.01). Created with Biorender.com

In a next step, we investigated the effect of the different diets on the immune cell composition of both mouse strains. For that, mice were sacrificed and blood, spleen, and gut collected for flow cytometry analysis. Due to the known role of the VDR and VD3 in stabilizing the gut epithelial barrier function, the immune cell infiltration in the gut was evaluated. For that purpose, we isolated Peyer´s patches from the small intestine and lamina propria leucocytes from the colon and small intestine of both mouse strains. As observed in **Figure 3A**, a diet containing 1500 IU/kg VD3 led to a significant increase in the percentage of CD11b+ cells in blood (**Figure 3A**) and a similar trend was observed for BALB/c mice for diets containing 1500 and 5000 IU/kg VD3. No differences were found in the percentage of TCRβ+ cells in the blood of both mice strains (**Figures 3A, B**). Regarding the spleen (**Figures 3C, D**) the same increase in CD11b+ cells was observed in both mouse strains. An increase of percentage of TCRβ + cells was observed only in C57BL/6 but not in BALB/c mice. In the gut, an impact of VD3 feeding was observed in the Peyer´s patches of BALB/c mice (**Figures 3E, F**). Again, higher VD3 levels in the diet lead to higher percentage of CD11b+ cells. Regarding TCRβ + cells, although a decrease was observed with 1500 IU/kg VD3, feeding with 5000 IU/kg VD3 led to a significant increase of TCRβ + cells to similar levels as 400 IU/kg VD3. Colon lamina propria leucocytes (LPL) were also modulated by different VD3 diets in both mice strains (**Figures 3G, H**). However, we observed an increase in the percentage of CD11b+ cells with 5000 IU/kg VD3 for C57BL/6 but not BALB/c mice. On the other hand, TCRβ + cells were only increased in BALB/c mice receiving 5000 IU/kg VD3 compared to 1500 IU/kg VD3 and no modulation was found for C57BL/6 mice. The amount of CD19+ cells was also evaluated (**Supplementary Figure S1**) and no significant changes were detected in the blood, spleen or colon LPL. However, mice receiving 5000 IU/kg VD3 had lower percentages of CD19+ cells in Peyer´s patches compared with mice receiving 1500 IU/kg VD3. No significant changes were detected in small intestine LPL (data not shown). Overall VD3 containing diets seem to mainly affect myeloid cells but not lymphocytes under steady state conditions in both mice strains.

### 3.4 VDR quantification in the gut of C57BL/66 and BALB/c mice

**Figure 4:**
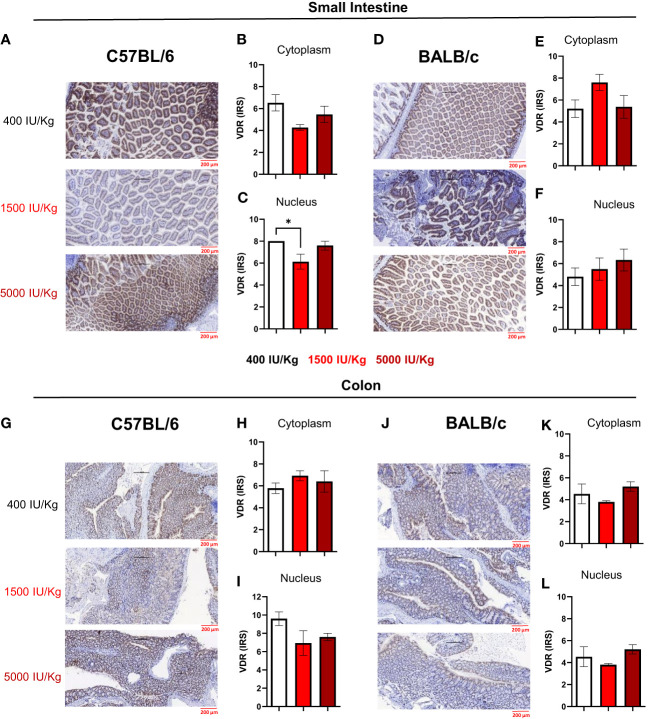
Immunoreactive score of VDR in the gut of C57BL/6 and BALB/c mice. Immunoreactive score of VDR immunostaining reveals slight changes in VDR expression after feeding different Vitamin D3 diets. **(A)** Representative staining of VDR in the small intestine of C57BL/6 mice fed with 400, 1500 and 5000 IU VD3. **(B, C)** display the mean IRS in both cytoplasm and nudeus of C57BL/6 mice. **(D)** Representative nucleus staining of VDR in the small intestine of BALB/c mice fed with 400, 1500 and 5000 IU VD3. **(E, F)** Display the mean IRS in both cytoplasm and nucleus of BALB/c mice. **(G)** Representative staining of VDR in the colon of C57BL/6 mice. **(H, I)** Display the mean IRS in both cytoplasm and nucleus of C57BL/6 mice. **(J)** Representative staning of VDR in the colon of BALB/c mice. **(K, L)** Display the mean IRS in both cytoplasm and nucleus of BALB/c mice. Created with Biorender.com

In a next step, we quantified VDR expression in the nucleus and cytoplasm of epithelial cells in the small intestine and colon of both mouse strains using the semi-quantitative immunoreactive score (IRS). **Figure 4A** displays a representative staining of the small intestine for C57BL/6 mice fed with the different diets. As observed in **Figure 4B**, 1500 IU/kg VD3 led to a slight decrease in the VDR IRS in the cytoplasm, and a significant lower IRS score was detected in the nucleus compared with 400 IU/kg VD3 (**Figure 4C**). **Figure 4D** displays a representative staining of the small intestine for C57BL/6 mice fed with the different diets. As observed in **Figures 4E** and **F**, no significant changes were observed for BALB/c mice. **Figure 4G** displays a representative staining of VDR in the colon of C57BL/6 mice, for all the diets. No differences were observed in the cytoplasm or nucleus (**Figures 4H, I**). **Figure 4J** displays a staining from the colon of BALB/c mice and similar to C57BL/6, no significant changes were observed upon feeding the mice with the different diets neither in cytoplasm nor in nucleus (**Figures 4K, L**). The VDR quantification on mRNA level was also performed (**Supplementary Figure S2**). Here we observed an opposite regulation in the small intestine of C57BL/6 mice vs BALB/c for the diets 400 IU/kg vs 5000 IU/kg VD3. Although a downregulation was observed for increasing VD3 content in the diet of C57BL/6 mice (**Supplementary Figure S2A**), in BALB/c we found that feeding the mice with 5000 IU/kg VD3 leads to an upregulation of VDR at mRNA level (**Supplementary Figure S2B**).

### 3.5 Vitamin D3 modulates Reg3γ and Batf gene expression in the gut of C57BL/6, but not BALB/c mice

**Figure 5:**
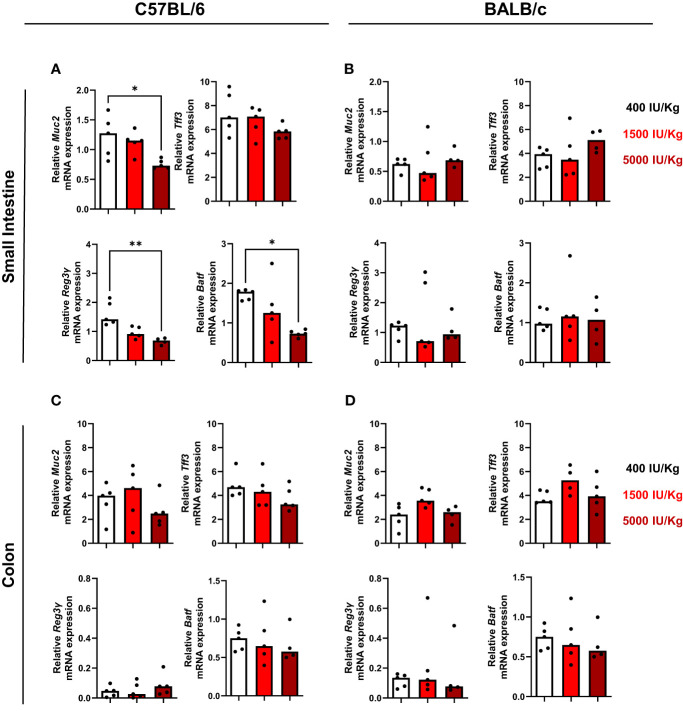
Vitamin D modulates Reg3γ and Batf gene expression in the gut of C57BL/6, but not BALB/c mice. qRT-PCR analysis of muc2, upper left panel, tff3, upper night panel, Reg3y bottom left panel and Batf, bottom right in the small intestine of C57BI/6 **(A)** and BALB/c **(B)** mice fed with different vitamin D3 diets. In **(C, D)** the same genes were analysed in the colon of the mice. Displayed is the median. Each dot represents one individual mouse. Statistical analysis was performed using one-way ANOVA, followed by Kruskal-Wallis test (*p≤0.05, **p≤0.01). Created with Biorender.com

Since it is known that VD3 influences intestinal homeostasis (9, 28), we investigated the expression of muc2, a mucin that forms the skeleton of intestinal mucus and protects the intestinal tract. Furthermore, the intestinal trefoil factor 3 (Tff3) expression was also investigated. Muc2 and Tff3 are co-produced and secreted by goblet cells of the small and large intestine. It was already demonstrated that dietary VD3 depletion leads to disrupted mucus barrier in the gut of mice and low Muc2 expression. As observed in **Figure 5A**, higher amounts of VD3 in the diet led to a decrease in Muc2 expression in the small intestine of C57BL/6, although no significant changes were detected for Tff3. We also analyzed the number of goblet cells in the gut of both mouse strains and a diet with 1500 IU/kg VD3 by trend decreased the number of goblet cells in C57BL/6. No alterations were detected for BALB/c mice (**Supplementary Figure S3**). VD3 is also known to modulate epithelial antimicrobial proteins (AMPs) which comprise a protein families, including defensins, S100 proteins, cathelicidins and C-type lectins (such as regenerating islet-derived protein (REG) family). Therefore, the expression of Reg3γ in the gut of mice fed a diet with different VD3 levels was also investigated. As observed in **Figure 5A** (left lower panel), increasing amounts of VD3 in the diet led to a decrease in Reg3γ expression, with expression being significant lower in mice receiving 5000 IU/kg VD3 compared to 400 IU/kg VD3. Several studies demonstrated the impact of vitamin D3 on IL-17 producing cells (29, 30). Based on this, the expression of AP-1 transcription factor basic leucine zipper transcription factor ATF-like (BATF), known to be a critical regulator of Th17 development, was also analyzed. Higher VD3 levels in the diet led to a decrease in Batf gene expression in the gut of C57BL/6 mice (**Figure 5A**, lower panel). The same genes were analyzed in BALB/c mice (**Figure 5B**) and in contrast to C57BL/6 mice, no regulation was detected by different VD3 diets. All genes were also analyzed in the colon of both mice strains (**Figures 5C, D**). No significant changes were detected in the colon of both mouse strains, with the exception of Batf, whose expression was again suppressed in C57BL/6 mice receiving the 5000 IU/kg VD3 diet (**Figure 5C**, lower right panel).

### 3.6 Microbiome differences in the gut of C57BL/6 and BALB/c mice fed with diets containing different vitamin D3 levels

**Figure 6:**
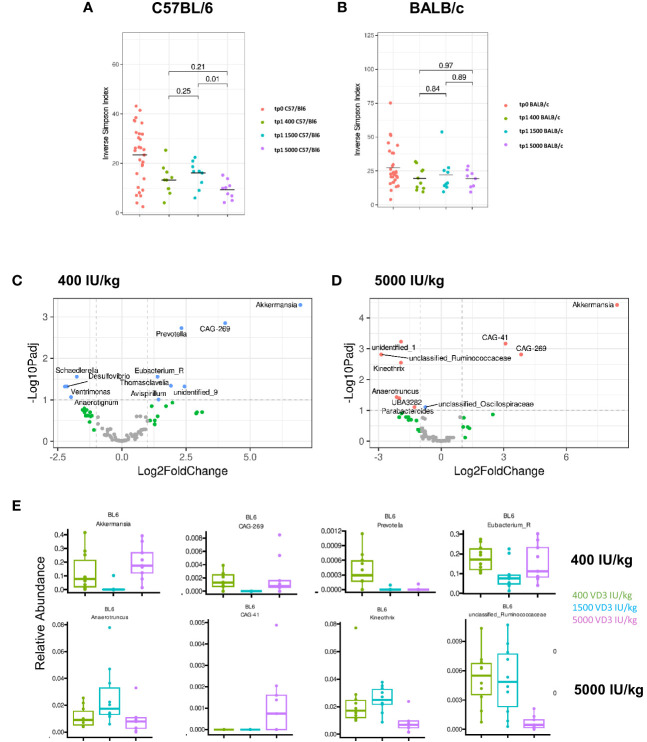
Diversity and compositional differences in the gut microbiome of C57BL/6 and BALB/c mice fed with diets containing different vitamin D3 levels. 16S rRNA gene sequencing analysis of C57BL/6 and BALB/c mice fed with different vitamin D3 diets. On **(A, B)** the inverse Simpson-index is depicted for C57BL/6 and BALB/c respectively. Volcano plots show log2 fold changes of genus-level bacterial abundances in the C57BL/6 1500 VD3 diet group compared with 400 IU VD3 **(C)** and 5000 IU VD3 **(D)** color code: grey = below p-value cutoff (01) and log2 fold-change cutoff (2); green = not significant, but above log2FC cutoff; blue = significant, but below log2FC cutoff; red = significant and above log2FC cutoff. On **(E)**, the relative abundance of selected genera is depicted, using the 1500 IU VD3 diet as reference compared with the 400 IU VD3 diet (upper panel) and 5000 IU VD3 (lower panel). Created with Biorender.com

There is growing evidence that the microbiome directly influences the inflammatory responses in the gut. Several factors contribute to microbiota differences and diversity, such as environmental pollution, stress, malnutrition, physical activity, gastrointestinal pathologies and the diet (31). VD3 has also been identified as a strong modulator of the gut microbiome (32). Since we observed changes in gene regulation by the different VD3 diets in the gut of C57BL/6 and BALB/c mice, we investigated whether the different diets affect the microbiome of both mouse strains in the same fashion. On day 0 and before the mice were put on different diets, we collected and analyzed the stool samples in order to establish the baseline composition of the mice. When analyzing the alpha diversity, as measured by the inverse Simpson-index, we observed that C57BL/6 mice lost diversity upon receiving the diets (**Figure 6A**). Although BALB/c mice followed the same trend, this loss was not as marked as in C57BL/6 mice (**Figure 6B**). Supplementing C57BL/6, but not BALB/c mice, with 5000 IU VD3/kg resulted in a significant loss of diversity compared with the standard 1500 IU VD3/kg. After dieting for 10 weeks, we observed that C57BL/6 mice lost abundance of some genera with the 1500 IU/kg VD3 diet (**Supplementary Figure S4**).

Since no significant changes were observed for BALB/c mice, we analyzed the alterations observed for C57BL/6 in more detail. The Volcano plots in **Figures 6C** and **D** show log2 fold-changes using the 1500 IU VD3/kg group as a reference. Genera on the right hand side of the plot are enriched in the respective group. We observed that there is a large overlap between the groups, as the composition of the 400 IU VD3 and 5000 IU VD3 dose is mostly similar. In both diets, CAG 269, an unclassified genus of the class Clostridia and *Akkermansia* are the genus most enriched compared with the standard 1500 IU VD3/kg diet. The relative abundance of selected species is presented in **Figure 6E**, with the standard VD3 diet used as a reference compared with the 400 IU VD3 diet (upper panel) and 5000 IU VD3 (lower panel).

This data show that the gut microbiota composition of C57BL/6 mice seem to be more responsive to different VD3 levels compared with BALB/c.

## 4 Discussion

The use of murine models represents an attractive tool for investigating pathophysiology of tumors, autoimmune and inflammatory diseases and for the development of novel therapies. Although such models represent useful tools to investigate and support basic hypotheses regarding underlying mechanisms causing the diseases, caution is necessary for the extrapolation of the data to the clinical setting. Human conditions are often characterized by heterogeneity of symptoms and disease development due to genetic, behavioural and environmental variations in the population. This heterogeneity coupled with species-specific differences regarding gene, protein expression and metabolic pathways, makes the use of animal models error-prone leading to incorrect assumptions.

Due to the observation that human and murine systems often behave differently, we asked if differences in VD3 responses could also be observed when analysing different mouse strains. Surprisingly, strong differences were detected in all analysed parameters between C57BL/6 and BALB/c mice.

In line, a work by Xue et al. (33) described that maternal vitamin D3 deficiency (VDD) induced different liver metabolic effects in different genetic background strains. Not only they observed that VDD was strongly dependent on the genetic strain, but also that each strain had different pathways altered.

In the present study, we also analysed differences between the mouse strains *in vitro*, and both cell strains behaved differently. In line with the published data (6, 34, 35) we observed by trend a modest modulation of IL-17, IFNγ and IL-10 cytokine production. Suppression of IFNγ secretion was only observed in C57BL/6 CD4+ T cells treated with 1,25(OH)2D3. In contrast, in the study by Staeva-Vieira et al. (35), the authors observed a downregulation of IFNγ secretion when culturing CD4+ T cells from BALB/c mice. However, the strongest effect was observed when T cells where cultured under Th1 polarizing conditions and the authors used an almost five times higher concentration of 1,25(OH)2D3, (24 nM) than the present study. Of course, we cannot exclude that higher concentrations would suppress cytokine secretion. However, even considering the possible autocrine production of 1,25(OH)2D3, it does not seem plausible to expect that a local production of 1,25(OH)2D3 will reach concentrations higher than 5 nM.

IL-17 was not significantly modulated in the presence of 1,25(OH)D3 in both murine strains in contrast published data described (34). In this study, Palmer and colleagues investigated the impact of 1,25(OH)2D3 on the development of CD4 effector cells, polarized under specific conditions. The authors observed that different CD4 T cell subtypes expressed different levels of the VDR. Based on these data, it would be interesting to analyse whether T cells derived from different mouse strains express different levels of VDR, or the retinoid X receptor (RXR) as VDR binds to the DNA as heterodimer with its partner RXR (36).

Regarding IL-10 secretion, we observed opposite trends upon addition of 1,25(OH)2D3 to T cell cultures. While in C57BL/6 mice a downregulation was observed, the opposite seems to occur in BALB/c cultures, in line with human data (7, 37). As different genes are regulated in different ways by VD3 in the 2 murine strains, it is unlikely that the VDR is not present but suggests that other transcription factors and cofactors are strain specific expressed which might explain the differential response to 1,25(OH)2D3.

The *in vivo* data also provided interesting differences between the two analysed mice strains. Feeding the mice with the same diet, C57BL/6 mice achieved higher plasma 25(OH)D3 levels, indicating that this strain could be more sensitive to vitamin D3 supplementation. In line, Misharin and colleagues (38) found that 1,25(OH)2D3 levels were higher in C57BL/6 than BALB/c mice. Consistent with the strain differences for serum 1,25(OH)2D3 levels, mRNA analysis for kidney Cyp27b1 (the enzyme responsible for catalysing the conversion of 25(OH)D3 to 1,25(OH)2D3) revealed that C57BL/6 mice had about 17 times higher Cyp27b1 vs. BALB/c mice.

When we analysed immune cells in the different compartments (blood, spleen and gut) we observed that the immune cell distribution in both C57BL/6 and BALB/c mice changed in response to the different VD3-fortified diet. From the analysed cell types, CD11b, a marker of myeloid cells, was most sensitive to changes in VD3 diets, indicating that myeloid cells represent a primary target of VD3. This could probably be explained by the fact that myeloid cells, such as monocytes, express the VDR under steady state conditions, while T cells need to be activated in order to express the VDR. Therefore, it can well be that under inflammatory conditions more differences would be observed in the T cell compartment.

As expected, the VDR was strongly expressed in gut epithelial cells. We observed a modest modulation in the small intestine and colon of C57BL/6 mice and in the small intestine of BALB/c mice fed with higher amounts of VD3. Since we performed the analysis under steady-state conditions, no strong differences were expected. Nevertheless, when looking at the absolute VDR mRNA expression in the gut of both mice strains, we observed that C57BL/6 mice seem to express higher levels compared to BALB/c mice (**Supplementary Figure S2**). Analysing the data from the 1500 IU/Kg VD3 diet, which is considered to be the “normal” diet for a mouse, VDR expression in the small intestine is about 1,9x higher in C57BL/6 compared with BALB/c mice, what could partially explain a stronger response of C57BL/6 epithelial cells to VD3. This might hold true for the gut, however regarding the response in other compartments such as blood or spleen, absorption in the gut and VD3 conversion may also play a role and should be investigated.

It is discussed whether VD3, like other fat-soluble nutrients, is absorbed by passive diffusion or shares absorption pathways with the structurally-related cholesterol. Rebout et al. (39) suggest that VD3 intestinal absorption also involves cholesterol transporters such as Scavenger Receptor class B type I, CD36 or Niemann-Pick C1 Like. Interestingly, genetic variations at the enterocyte level seem to determine interstrain variations in cholesterol absorption (40) and the level of biliary Niemann-Pick type C 2 (NPC2), a cholesterol-binding protein, is 3-fold higher in C57BL/6 mice compared to the BALB/c strain (41). Therefore, strain-specific differences in cholesterol transport may also affect VD3 absorption and could explain different 25(OH)D3 levels in these mouse strains.

Overall, since C57BL/6 mice are known to be prone to generate an inflammatory Th1- (and M1)- dominant immune response, one could argue that this mouse strain needs more counter regulation by VD3 compared with BALB/c that show a more Th2- (and M2)- phenotype.

Loss of the VDR and low circulating levels of 25(OH)D3 are observed in IBD, colitis and GvHD (8), highlighting the importance of VD3 in inflammatory conditions in the gut (42, 43). Due to the strong reported effects of vitamin D3 on gut physiology, we analysed different genes in order to identify possible VD3 targets in both strains.

Since mucins and antimicrobial peptides play an essential role in protecting the epithelium wall, we sought to investigate if differences were observed in the amount Muc2 and Reg3γ in both colon and small intestine. We observed a significant decrease of Muc2 at mRNA level in C57BL/6 mice fed a diet supplemented with 5000 IU/kg VD3. In line, Reg3γ levels were also downregulated in the small intestine of C57BL/6 mice, indicating that Reg3γ can be a direct or indirect target of VD3. As Muc2 is essential for epithelium wall protection and VD3 was already described as an important player for epithelial barrier maintenance, one would expect that higher VD3 in the diet would result in higher Muc 2 expression. The same holds true for Reg3γ, as it is important for the physical separation of microbiota from the host and thereby regulates the immune response. Probably, mucin and Reg3γ regulation under steady-state conditions is different from an acute inflammation, which should be investigated in further studies. On the other hand, one report (44) states that high dose (10.000 IU/kg) VD3 supplementation predisposes mice to more severe colitis, a condition accompanied by a loss in epithelial barrier integrity. In line, a suppression of mucin and Reg3γ was only observed in mice fed with the high 5000 IU/kg VD3 diet in our study.

BATF is a transcription factor whose upregulation was detected in patients with ulcerative colitis (UC) and colorectal cancer (CRC) (45). Furthermore, Batf is required for Th17 differentiation. Since IL-17, a cytokine mainly secreted by Th17 cells is also a VD3 target, we asked if VD3 could modulate Batf expression in the gut of mice. We observed that in both small intestine and colon, 5000 IU/kg VD3 was capable of supressing Batf mRNA expression in C57BL/6 mice. This could provide a possible mechanism by which VD3 is capable to suppress IL-17 expression. It would be interesting to examine IL-17 expression in the gut in further studies as our *in vitro* data did not reveal a clear regulation of IL-17 in T cells by 1,25 (OH)2D3.

Gene regulation by VD3-fortified diets was only observed in C57BL/6. At a first glance, one could argue that C57BL/6 achieved higher serum 25(OH)D3 levels, therefore, more differences were detected in C57BL/6 mice. However, gene regulation by VD3 was only observed at a dose of 5000 IU/kg VD3 but, no differences were found in circulating 25(OH)D3 levels between the 1500 IU/kg and 5000 IU/kg diet. Nevertheless, one cannot exclude that the circulating 25(OH)D3 levels probably do not reflect the local amount of 25(OH)D3 and it can well be that the concentration achieved in the tissue is higher for mice receiving 5000 IU/kg VD3. Furthermore, C57BL/6 mice seem to have higher basal levels of the VDR, at least at mRNA level. This could also in part explain the observed stronger modulation by VD3 in this mouse strain.

In a study by Mukhopadhyay and colleagues (46), the authors investigated whether C57BL/6 or BALB/c mice had different susceptibility to colitis. BALB/c (Th2 biased) resisted severe inflammation by modulating the host´s inflammatory, metabolic and gut microbiome profile whereas in C57BL/6 mice, DSS induction resulted in more inflammation with an upregulation in amino acid and lipid metabolism, Toll-like receptor (TLR) and NOD-like receptor activation compared with BALB/c mice. The observed higher VDR expression in the gut of C57BL/6 compared with BALB/c could represent a possible compensatory mechanism in which VDR is upregulated in Th1 biased mice to achieve gut homeostasis.

The microbiome profile is also known to play an important role in the outcome of several pathologies and their response to treatment. In the same study, the researchers found that both murine strains lost microbial diversity upon DSS colitis induction. However, this loss seemed to be permanent for C57BL/6 mice, whereas the diversity was restored in BALB/c mice.

Currently, it is well acknowledged that VD3 is capable of modulating the microbiome composition. Studies in men and mice assessed the impact of VD3 deficiency or VD3 supplementation on the microbiota composition. We also detected differences in the microbiome of both strains upon VD3 dieting. C57BL/6 mice were more sensitive to the different VD3 diets than BALB/c mice. By analysing the inverse Simpson Index, we observed a drop in alpha diversity of C57BL/6 mice upon receiving the VD3 diets, which was not observed for the BALB/c mice. Although all diets showed a marked decrease in diversity, 5000 IU VD3 diet resulted in the strongest effect. This data goes in line with the work of Ghaly and colleagues (44), where they also observed that high VD3 supplementation resulted in the loss of microbial diversity. These results suggest that an optimal dose of VD3 is necessary to achieve a balanced microbiome.

Looking at the microbiome composition at genus level, our data show a resemblance in 400 IU/kg and 5000 IU/kg VD3 diets with a dominance of the genera, *Akkermansia and* CAG-269, a unclassified genus of the class Clostridia. These specimens are very low in abundance in mice receiving 1500 IU/kg VD3 diets.

*Akkermansia muciniphila*, a mucin-degrading species, seems to be responsive to diet and VD3 variations however, there is some controversy whether *Akkermansia* represents a health-promoting beneficial taxa (47). In healthy humans, VD3 supplementation upregulated *Akkermansia* (48). In contrast, Zhu and colleagues analyzed the composition of the gut microbiota in 1,25(OH)2D3-deficient mice where deficiency led to a higher *Akkermansia muciniphila* abundance, thinner mucus layer and increased bacterial translocation. The authors described no impact on goblet cells and suggested that 1,25(OH)2D3 limits colonization of *Akkermansia. muciniphila* (49). These data correspond to our findings in C57BL/6 mice with similar number of goblet cells (**Supplementary Figure S3**) but a significant higher *Akkermansia* prevalence in VD3-deficient diet compared to VD3 standard diet. Conflicting data were published in BALB/c mice fed a VD3 deficient diet. Here, *Akkermansia muciniphila* was decreased significantly in mice fed with a VD3-deficient diet, especially when combined with a high fat diet (17). In our feeding experiments, no significant VD3-induced alterations were found in BALB/c mice. Overall it seems that *Akkermansia* in principle can be regulated by VD3, however its impact is strongly dependent on species, strain and diet composition.

Overall, a VD3 fortified diet seems to have more modulatory effects in the gut of C57BL/6 mice compared to BALB/c, that could at least partially be explained by the higher VDR expression found in the gut of C57BL/6 mice. VDR upregulation could serve as mechanism to insure an immunological balance in the gut. The present study demonstrates several strain-specific effects of VD3 indicating that one has to be cautious with testing and extrapolating effects of VD3 from murine data for the clinical setting.

## Data availability statement

The original contributions presented in the study are included in the article/**Supplementary Materials**, further inquiries can be directed to the corresponding author/s.

## Ethics statement

The animal study was approved by Government of Lower Frankonia (Regierung von Unterfranken/55.2.2-2532-2-1220-31). The study was conducted in accordance with the local legislation and institutional requirements.

## Author contributions

LS: Investigation, Writing – review & editing. SG: Investigation, Writing – review & editing. KR: Data curation, Investigation, Writing – review & editing. AH: Data curation, Investigation, Methodology, Writing – review & editing. MA: Investigation, Methodology, Writing – review & editing. NB: Investigation, Writing – review & editing. AP: Investigation, Writing – review & editing. GS: Investigation, Writing – review & editing. KH: Methodology, Writing – review & editing. AG: Methodology, Writing – review & editing. CA: Methodology, Writing – review & editing. FP: Investigation, Writing – review & editing. MB-H: Methodology, Writing – review & editing. HB: Funding acquisition, Writing – review & editing. PH: Methodology, Writing – review & editing. WH: Funding acquisition, Writing – review & editing. EH: Methodology, Writing – review & editing. KP: Conceptualization, Methodology, Writing – review & editing. MK: Conceptualization, Funding acquisition, Project administration, Supervision, Writing – review & editing. CM: Conceptualization, Data curation, Investigation, Methodology, Project administration, Supervision, Writing – original draft.
