# Supplementary material for: Strain specific differences in vitamin D3 response: impact on gut homeostasis
Source: Front Immunol. 2024 Mar 1;15:1347835. doi: 10.3389/fimmu.2024.1347835 (PMC10943696; doi:10.3389/fimmu.2024.1347835)
Supplement: Supplementary file 1 [file DataSheet_1.pdf]

Figure S1. Amount of CD19+ Cells in C57BL/6 and BALB/c mice in different compartments

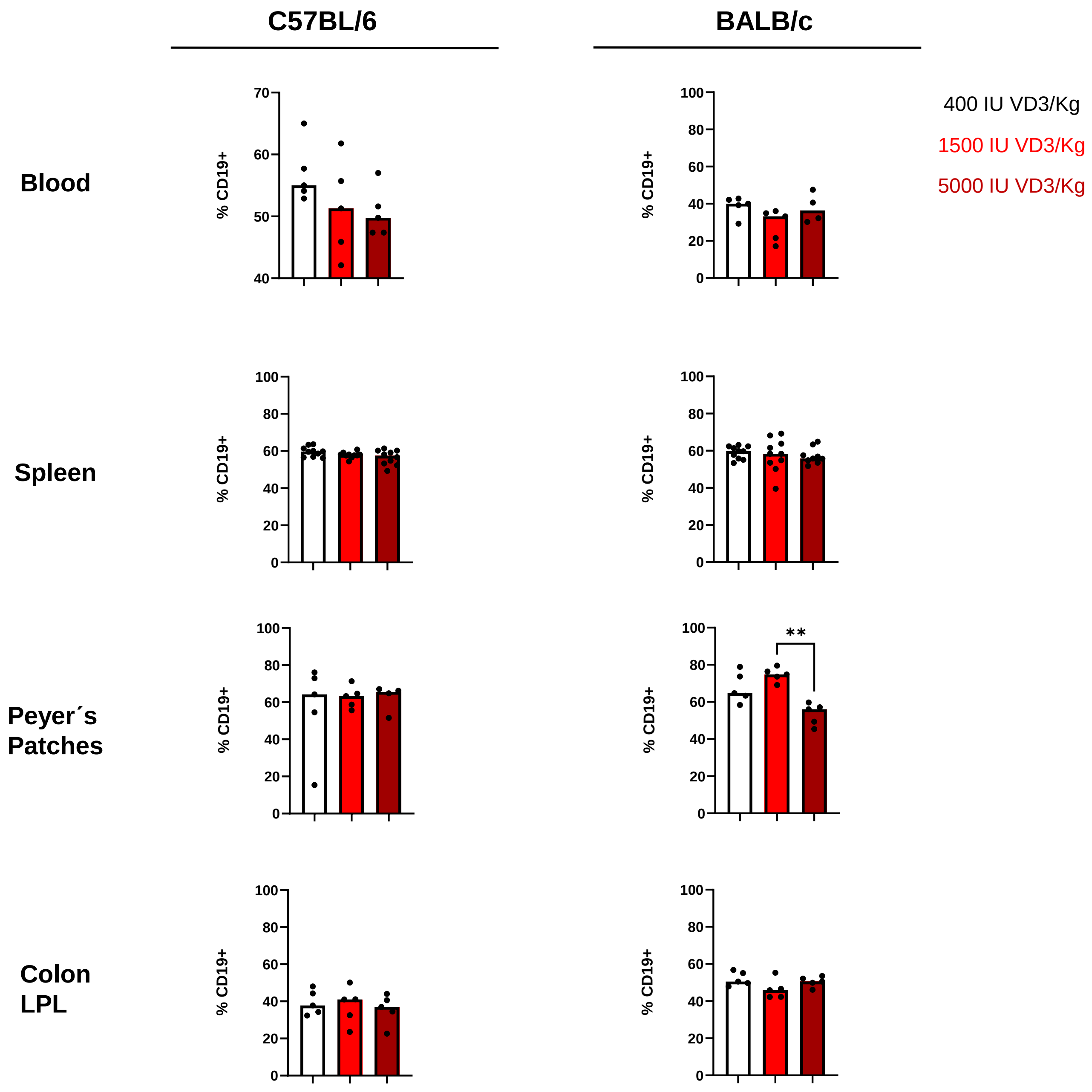

Figure S1. Percentage of CD19+ cells in blood, spleen and gut of C57BL/6 and BALB/c mice. Displayed is the median. Each dot represents one individual mouse. Statistical analysis was performed using one-way ANOVA, followed by Kruskal-Wallis test (*p ≤ 0.05, ** p ≤ 0.01).

Figure S2. mRNA VDR and CYP27A1 quantification in the gut of both C57BL/6 and BALB/c mice

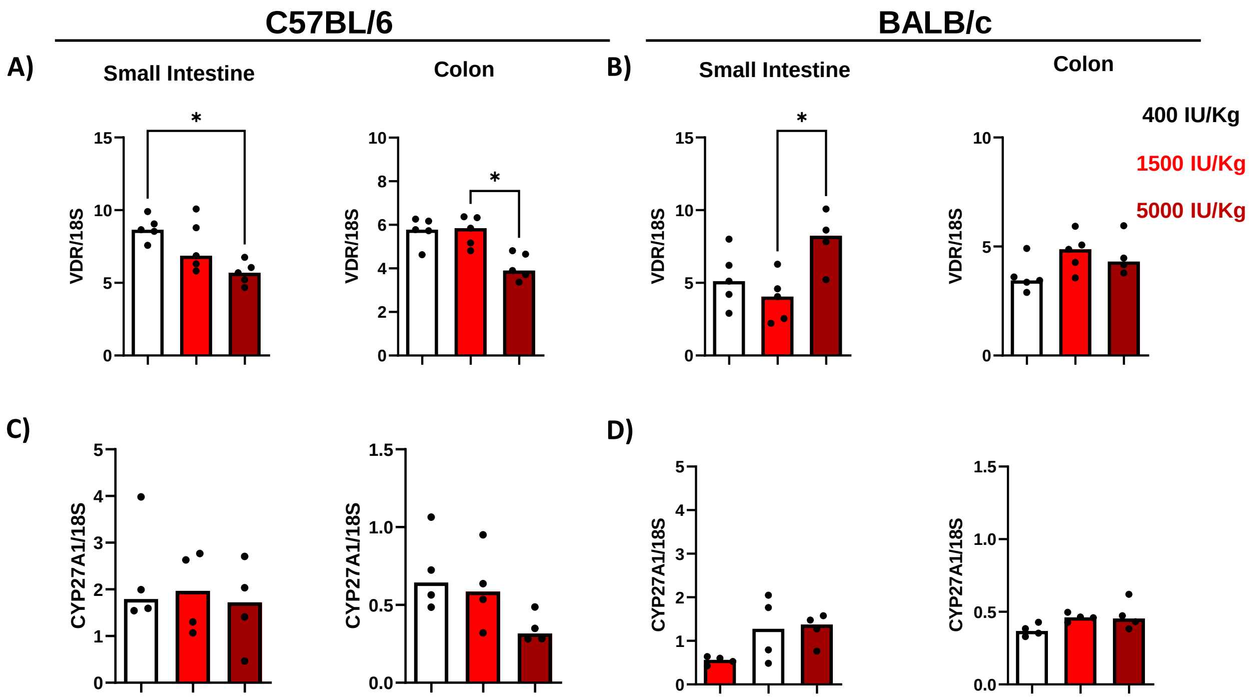

Figure S2. qRT-PCR analysis of VDR in the small intestine and colon of C57BL/6 (A) and BALB/c (B) mice fed with different vitamin D3 diets. In (C) and (D), CYP27A1 expression levels are shown. Displayed is the median. Each dot represents one individual mouse. Statistical analysis was performed using one-way ANOVA, followed by Kruskal-Wallis test (*p ≤ 0.05, ** p ≤ 0.01).

Figure S3. Goblet cell quantification in the small intestine of both C57BL/6 and BALB/c mice

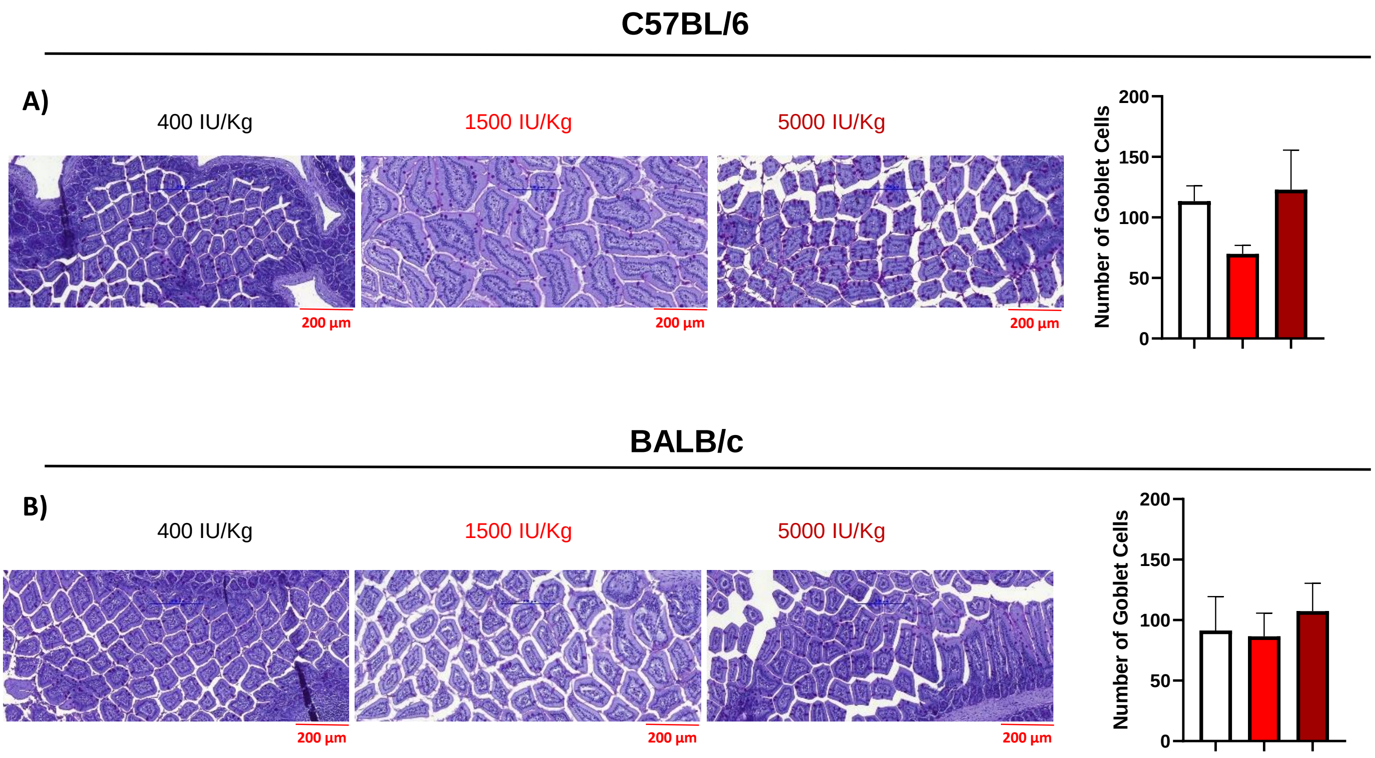

Figure S3. Goblet cell quantification in the small intestine of C57BL/6 and BALB/c mice. Goblet cells were identified using PAS staining.

Figure S4. Microbiome differences in the gut of C57BL/6 and BALB/c mice fed with diets containing different vitamin D3 levels

A)

C57BL/6

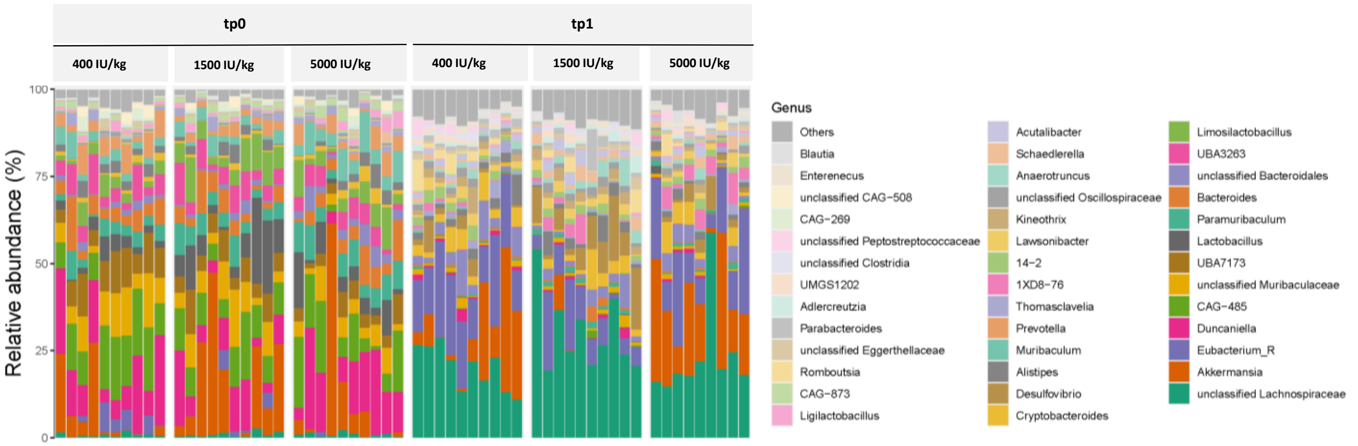

B)

BALB/c

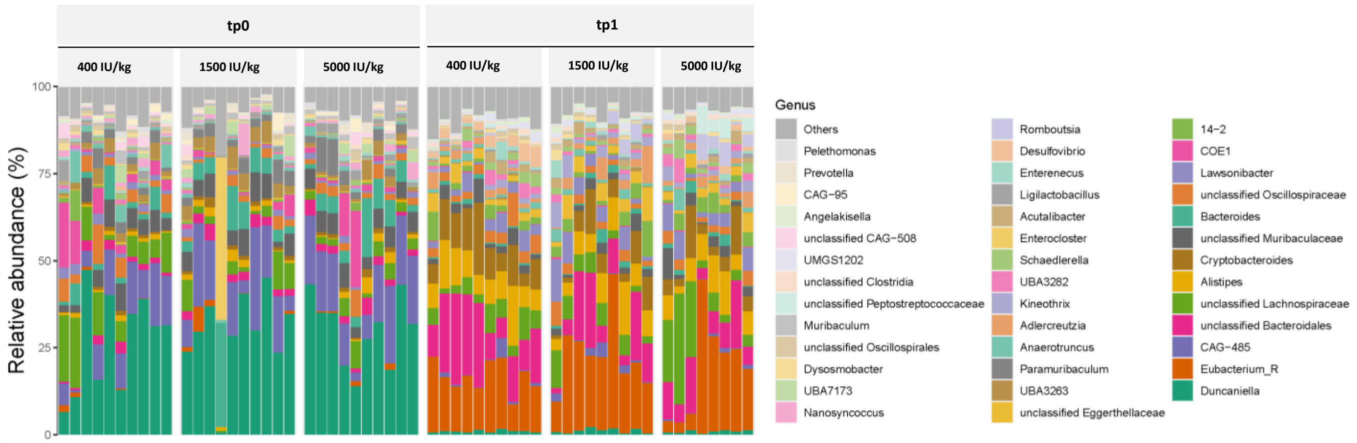

Figure S4. Effects on changes of gut microbiota composition in mice by feeding them different vitamin D3 containing diets. Compositional analysis of C57BL/6 (A) and BALB/c (B) gut microbiota in the groups using 16 S rRNA sequencing. Depicted is the relative abundance (%) of bacterial genus.
